# A Spatiotemporal Solution to Control COVID-19 Transmission at the Community Scale for Returning to Normalcy: COVID-19 Symptom Onset Risk Spatiotemporal Analysis

**DOI:** 10.2196/36538

**Published:** 2023-01-06

**Authors:** Chengzhuo Tong, Wenzhong Shi, Anshu Zhang, Zhicheng Shi

**Affiliations:** 1 Otto Poon Charitable Foundation Smart Cities Research Institute and Department of Land Surveying and Geo-Informatics, The Hong Kong Polytechnic University Hong Kong China (Hong Kong); 2 Research Institute for Smart Cities, School of Architecture and Urban Planning, Shenzhen University Shenzhen China

**Keywords:** return to normalcy, precise prevention and control, risk prediction, COVID-19 symptom onset, symptom, COVID-19

## Abstract

**Background:**

Following the recent COVID-19 pandemic, returning to normalcy has become the primary goal of global cities. The key for returning to normalcy is to avoid affecting social and economic activities while supporting precise epidemic control. Estimation models for the spatiotemporal spread of the epidemic at the refined scale of cities that support precise epidemic control are limited. For most of 2021, Hong Kong has remained at the top of the “global normalcy index” because of its effective responses. The urban-community-scale spatiotemporal onset risk prediction model of COVID-19 symptom has been used to assist in the precise epidemic control of Hong Kong.

**Objective:**

Based on the spatiotemporal prediction models of COVID-19 symptom onset risk, the aim of this study was to develop a spatiotemporal solution to assist in precise prevention and control for returning to normalcy.

**Methods:**

Over the years 2020 and 2021, a spatiotemporal solution was proposed and applied to support the epidemic control in Hong Kong. An enhanced urban-community-scale geographic model was proposed to predict the risk of COVID-19 symptom onset by quantifying the impact of the transmission of SARS-CoV-2 variants, vaccination, and the imported case risk. The generated prediction results could be then applied to establish the onset risk predictions over the following days, the identification of high–onset-risk communities, the effectiveness analysis of response measures implemented, and the effectiveness simulation of upcoming response measures. The applications could be integrated into a web-based platform to assist the antiepidemic work.

**Results:**

Daily predicted onset risk in 291 tertiary planning units (TPUs) of Hong Kong from January 18, 2020, to April 22, 2021, was obtained from the enhanced prediction model. The prediction accuracy in the following 7 days was over 80%. The prediction results were used to effectively assist the epidemic control of Hong Kong in the following application examples: identified communities within high–onset-risk always only accounted for 2%-25% in multiple epidemiological scenarios; effective COVID-19 response measures, such as prohibiting public gatherings of more than 4 people were found to reduce the onset risk by 16%-46%; through the effect simulation of the new compulsory testing measure, the onset risk was found to be reduced by more than 80% in 42 (14.43%) TPUs and by more than 60% in 96 (32.99%) TPUs.

**Conclusions:**

In summary, this solution can support sustainable and targeted pandemic responses for returning to normalcy. Faced with the situation that may coexist with SARS-CoV-2, this study can not only assist global cities in responding to the future epidemics effectively but also help to restore social and economic activities and people’s normal lives.

## Introduction

After more than a year of the COVID-19 pandemic, people are beginning to look forward to the return of normalcy in the forms of no masks, no isolation, and no social distancing [1]. A global normalcy index has been compiled by the Economist to measure the current daily activities relative to the prepandemic level in the 50 largest economies in the world [2]. The index comprises 8 indicators, split into 3 domains, as follows: transport and travel, recreation and entertainment, and retailing and work [2]. According to the data as of December 31, 2021, Hong Kong is at the top of the normalcy index [2]. Hong Kong is one of the most densely populated cities and ranks one of the top 5 territories for population density in the world. The high population density together with the highly developed international transportation networks make it vulnerable to importation as well as the local spread of SARS-CoV-2 variants [3-5]. Despite the severe challenges [6-8], Hong Kong’s figures on confirmed and fatal cases are among the lowest in the world [9]. Even in the face of the rapidly spreading variant of Omicron, the local epidemic situation in Hong Kong has remained stable, and no local cases have been recorded for 80 consecutive days [9,10]. Hong Kong’s economy is exhibiting major signs of recovery with people’s lives subsequently being back to normal [11].

It is of note that from the outbreak of the COVID-19 epidemic on January 18, 2020, only strict social distancing and crowd control measures were adopted in Hong Kong, as opposed to extreme measures, such as citywide lockdowns or curfews [12]. The overall aim of this new direction was to formulate sustainable and targeted pandemic responses and hence more effectively enable better control of the epidemic situations [13]. A spatiotemporal solution has been developed to enable the prediction of COVID-19 symptom onset risk and provide support for Hong Kong’s new direction of pandemic prevention.

The improved intercity-scale [14,15] and urban-community–scale [16] weighted kernel density estimation (WKDE) models have been applied to predict spatiotemporal COVID-19 symptom onset risk [17,18]. The above extended WKDE models provide competitive advantages based on high viral load around the date of symptom onset [19,20] as well as the delay between the onset dates and subsequent confirmation report dates [21-23]. Thus, the tracking of the COVID-19 symptom onset risk better reflects the COVID-19 transmission at the urban-community scale. However, the urban-community–scale WKDE model needs further improvement regarding the new normal of long-term coexistence with SARS-CoV-2 [24]. Consequently, the urban-community–scale WKDE model has been further enhanced by introducing the reproduction number for local cases, the number of passenger arrivals at all ports of entry, time-varying vaccination rate, and vaccination efficiency.

The enhanced urban-community–scale WKDE model has been used to predict the risk of COVID-19 symptom onset in each of the 291 tertiary planning units (TPUs) in Hong Kong. It generates the following urban-community–scale prediction results: the daily onset risk prediction results during the following few days and the daily onset risk prediction enhanced by the population distributions within 291 TPUs during the following few days. Hence, these applications are able to be implemented to support precise prevention and control as well as the recovery of socioeconomic activities in Hong Kong. A web-based visualization platform has been further developed to present the above prediction and application results. The study was conducted based on a spatiotemporal data set of 7184 local onset cases [25] with community-level locations in Hong Kong from January 18, 2020, to April 22, 2021, including, so far, all four epidemic waves of COVID-19 epidemics in Hong Kong [26].

## Methods

### Data Sources

In addition to asymptomatic cases, imported cases under mandatory quarantine, and cases with unknown location information, a total of 7184 local onset cases from January 18, 2020, to April 22, 2021, have been used in this study. These local onset cases include information regarding the date of the symptom onset and consequent report as well as the community-level location of these onset cases prior to diagnosis. Currently, the main COVID-19 vaccines used in Hong Kong are mRNA (BNT162b2) vaccines [27] and inactivated (CoronaVac) SARS-CoV-2 vaccines [28]. Thus, to measure the impact of vaccination on the COVID-19 epidemic, daily data [29] on people with full vaccination were selected, including the daily number of people receiving the second dose of the BNT162b2 vaccine [29] and the daily number of people receiving the second dose of the CoronaVac vaccine [29]. The daily traffic flow data covering all Hong Kong strategic routes from January 18, 2020, to April 22, 2021, were used in this study [30]. To enable the measurement of the risk of the imported cases, the daily number of passenger arrivals at all 16 ports of entry during the same period was also obtained [31]. Moreover, the reproductive number for local cases during the same period was generated by an enhanced Susceptible-Infectious-Removed model [22].

### An Enhanced Urban-Community–Scale WKDE Model for Predicting the Onset Risk of COVID-19 Symptoms

As a further development of the original urban-community–scale WKDE model, the enhanced urban-community–scale WKDE model was used in this study [14-16]. The details of this model are presented in Multimedia Appendix 1. Finally, the original onset risk prediction in each location was divided by the city’s maximum predicted risk, on a specific date, and thereby, standardized to a value between 0 and 1 [14-16]. Following the idea of a hit rate, the accuracy of the enhanced urban-community–scale WKDE model is set as the percentage of onset cases, on the date of prediction, that occur in the areas with predicted onset risk higher than 0.8 (known as hotspots) [14-16]. The population distributions in different communities were further used to enhance the original onset risk (details are presented in Multimedia Appendix 1).

### Ethical Considerations

For this study, no ethics approval was required because the granularity of the data was at the city level.

## Results

### Daily Spatiotemporal Risk Prediction of COVID-19 Symptom Onset by the Enhanced Urban-Community–Scale WKDE Model

**Figure 1:**
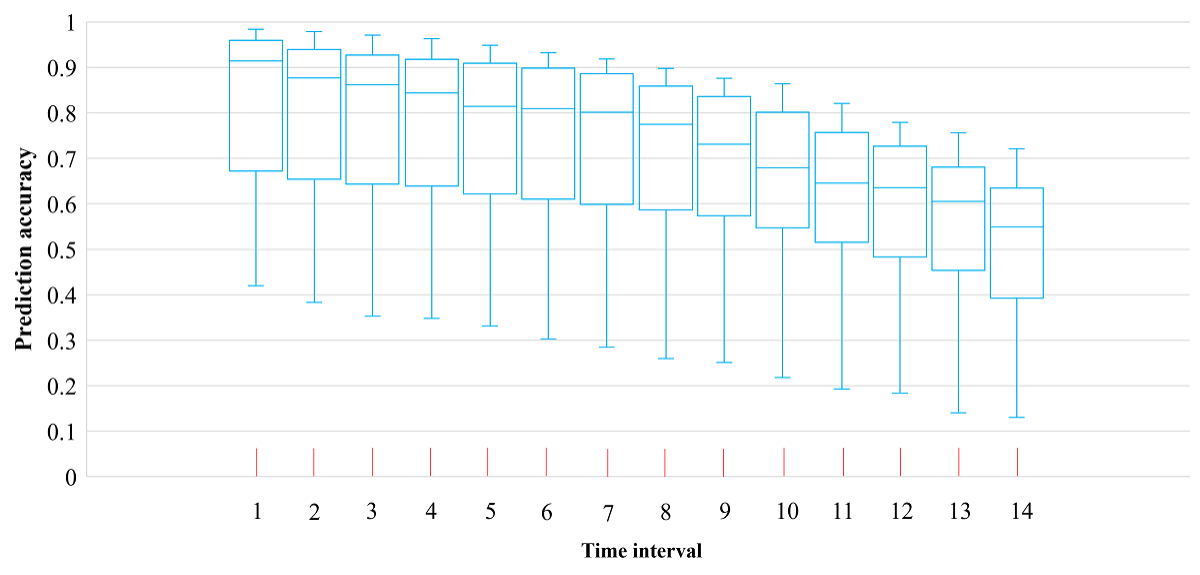
Accuracy of the predicted risk of COVID-19 symptom onset by the enhanced urban-community–scale weighted kernel density estimation model.

Based on the onset cases data in 291 TPUs of Hong Kong from January 18, 2020, to April 24, 2021, the daily spatiotemporal onset risk predictions of COVID-19 symptoms were obtained by the enhanced urban-community–scale WKDE model developed in this study. During the following first week after predicting the onset risk, the median prediction accuracy of the urban-community–scale WKDE model was over 80% (Figure 1). The prediction accuracy during the following second week, as possibly could be expected, was lower due to the accumulation of prediction errors over time.

**Figure 2:**
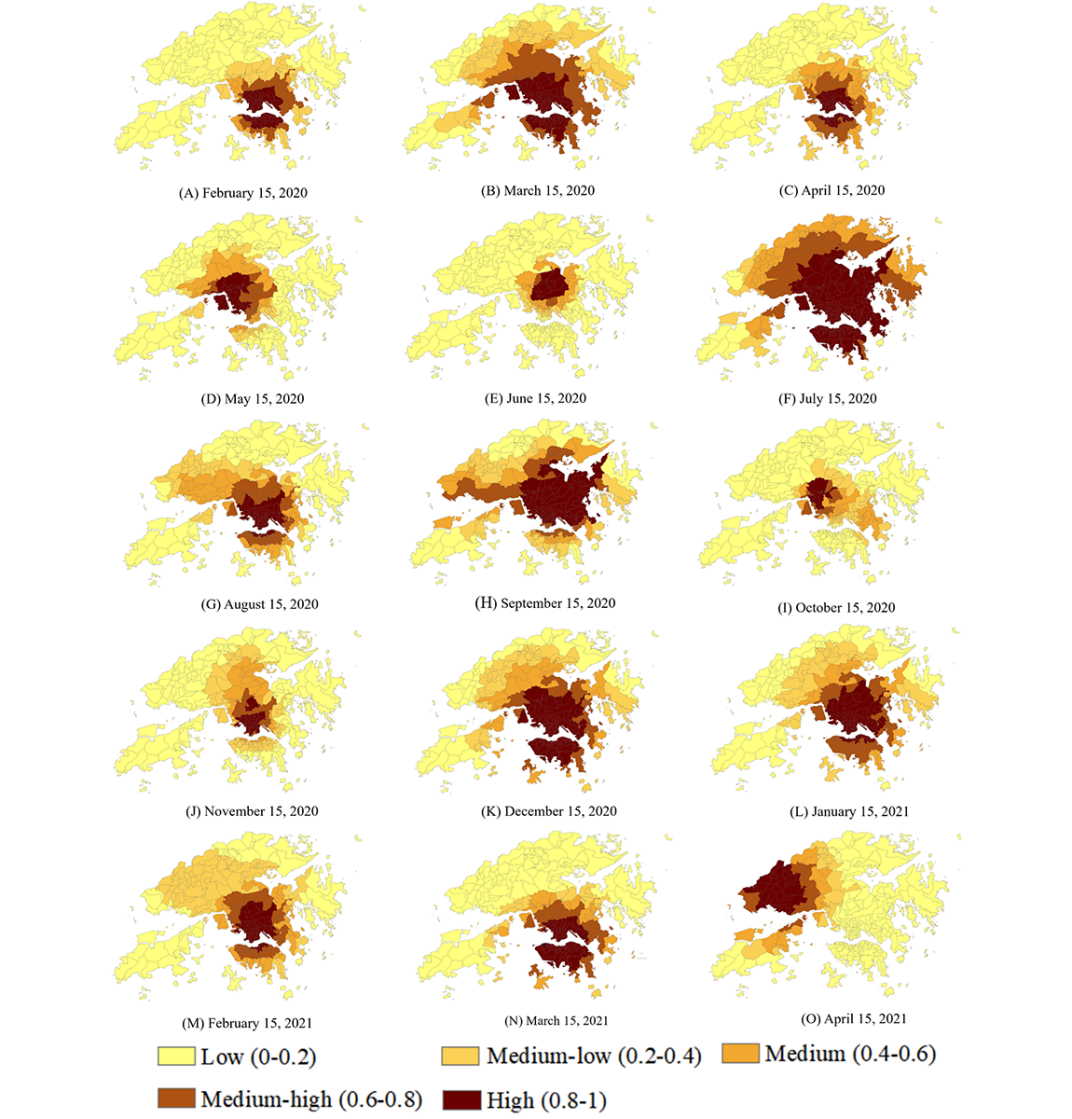
Predicted risk of original COVID-19 symptom onset risk across Hong Kong (A-O) in all four epidemic waves from January 18, 2020, to April 24, 2021.

In all four epidemic waves caused by different SARS-CoV-2 variants, the daily spatiotemporal distributions of urban-community–scale COVID-19 symptom onset risk from January 18, 2020, to April 24, 2021, were used to measure urban-community–scale heterogeneity (Figure 2). Communities with the onset risk higher than 0.8 were always concentrated in Central and Western, Wan Chai, Kowloon City, and Yau Tsim Mong, all of which are central shopping and commercial areas with a very high traffic flow. Conversely, the onset risk was relatively low in suburban communities, far away from the main transportation network. The Q statistic results conducted by Geodetector showed that the variations in mobility had a very strong effect on variations in onset risk in 291 TPUs, with a Q value of 0.98 (*P*=.005). Technological details of Geodetector is presented in Multimedia Appendix 1. Moreover, the trend on the daily regional shift regarding different onset risk levels could be visually displayed.

### Identification of High–Onset-Risk Communities in Three Epidemiologic Setting Scenarios

From the identified results of high–onset-risk communities based on the daily prediction of the original onset risk, the number of TPUs within high–onset-risk areas always only accounted for 2%-25% under the two epidemiologic setting scenarios of no cases and sporadic and clusters of cases. This enabled only the most stringent measures needing to be implemented in a very limited area within the high onset risk to reduce the impact on normal activities in other areas. In addition, such an identification of high–onset-risk communities also supports the allocation of limited COVID-19 medical supplies in Hong Kong.

**Figure 3:**
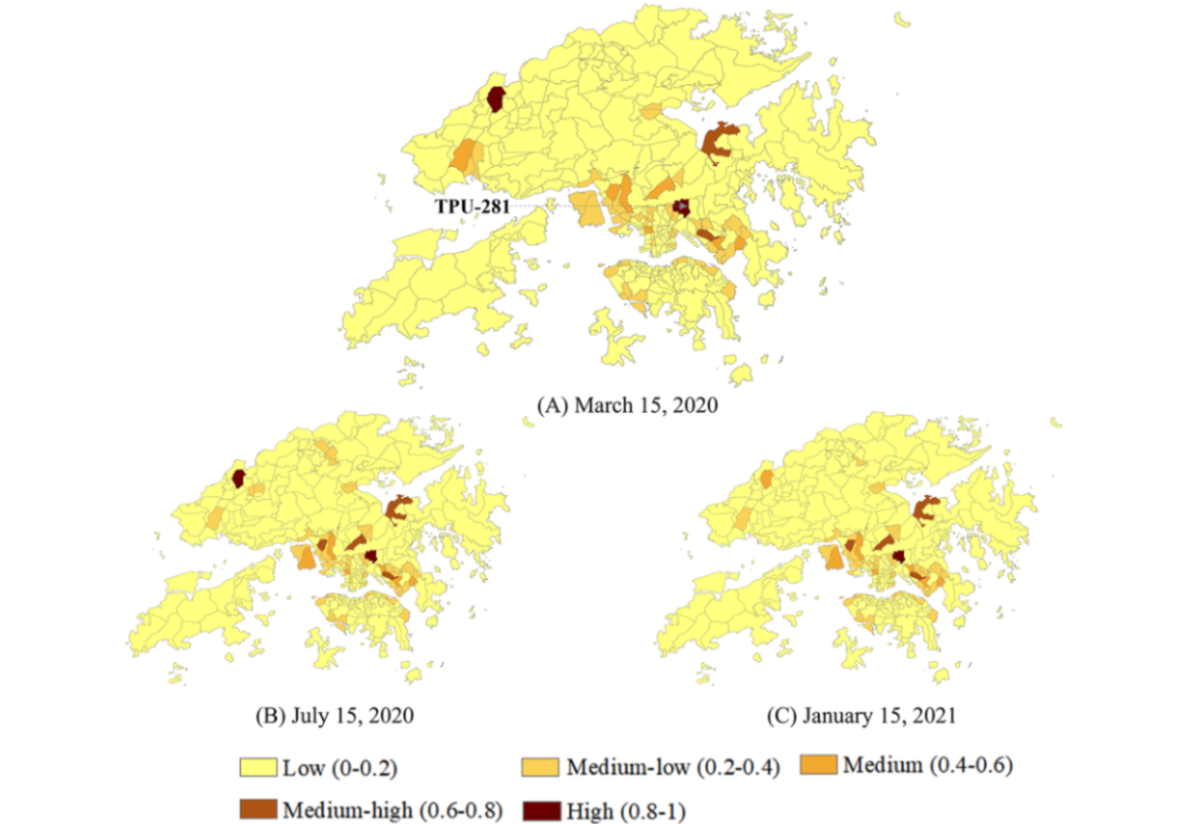
Predicted COVID-19 symptom onset risk enhanced by the population distributions within 291 tertiary planning units (TPUs) of Hong Kong (A-C).

However, in the community transmission scenario, the high–onset-risk communities identified directly from the original symptom onset risk prediction did not seem to be controlled in a small area. For example, on July 15, 2020—the peak of the third wave of the epidemic in Hong Kong—the high–onset-risk communities identified accounted for 155 (53.26%) of all 291 TPUs (Figure 2F). Limited COVID-19 medical supplies and human resources for health could not be allocated to the communities that needed them most. Based on the enhanced identification of high–onset-risk communities by population distributions, even during the peak period of the four epidemic waves, communities within the high onset risk could be reduced to a limited area (Figure 3A-C).

### Assistance in the Effect Analysis and Implementation of COVID-19 Response Measures

**Figure 4:**
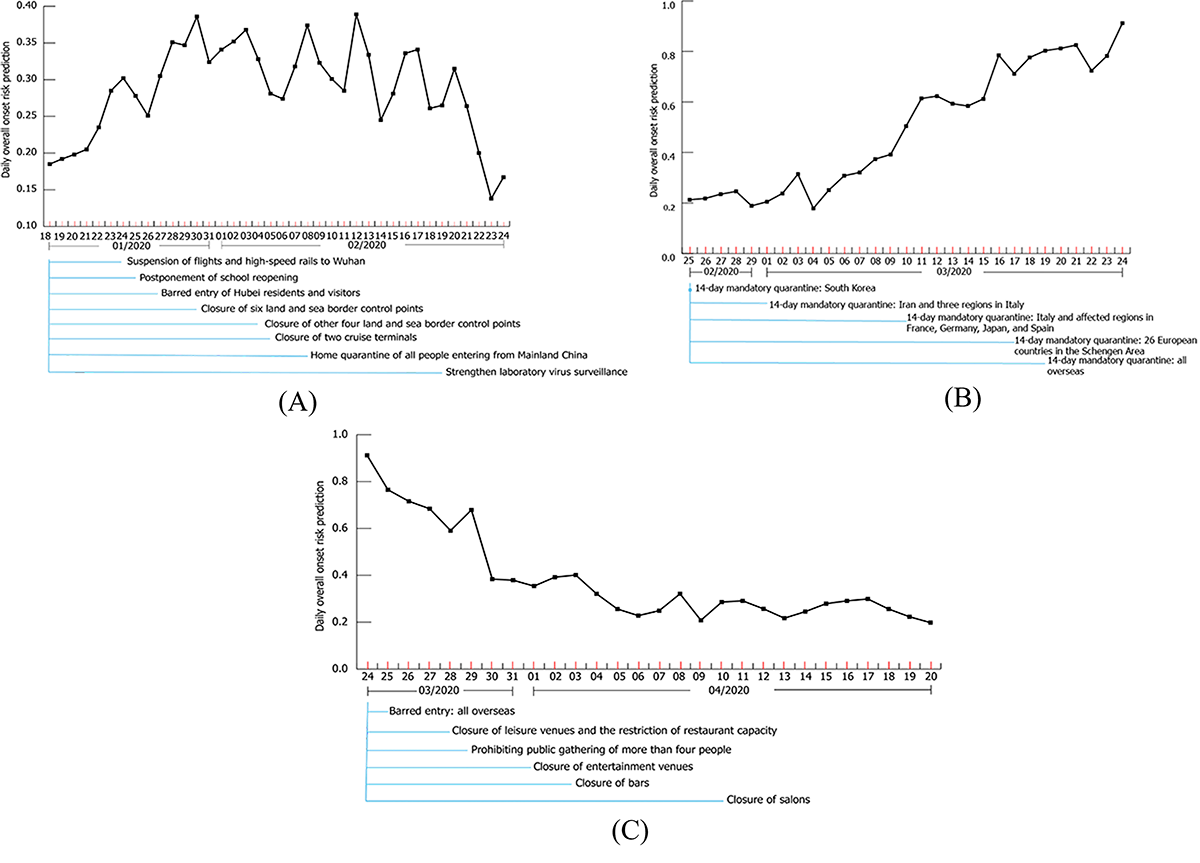
The average daily variation of the risk of COVID-19 symptom onset from January 18 to April 20, 2020; (A) average daily onset risk in all Hong Kong tertiary planning units (TPUs) from January 18 to February 24, 2020 (during this period, all COVID-19 response measures implemented were marked); (B) average daily onset risk in all Hong Kong TPUs from February 25 to March 24, 2020 (during this period, all COVID-19 response measures implemented were marked); (C) average daily onset risk in all Hong Kong TPUs from March 24 to April 20, 2020 (during this period, all COVID-19 response measures implemented were marked).

During the early stages of the epidemic, a series of previously untried COVID-19 response measures were implemented to suppress the spatiotemporal spread of SARS-CoV-2, including stringent border control measures to guard against imported cases and social distancing measures to prevent the local spread of the virus. Thus, it was necessary to evaluate the effect of these COVID-19 response measures on the spatiotemporal onset risk so as to adjust the COVID-19 response measures during subsequent stages. Thus, the effects of border control measures and social distancing measures already implemented in the first and second waves during the early stage of the epidemic were evaluated by the overall onset risk variations in 291 TPUs of Hong Kong within one week of the implementation (Figure 4). For border control measures, barred entry of all non-Hong Kong residents seemed to be the most effective during the peak period of the epidemic caused by imported cases. After its implementation, the overall onset risk was reduced by 28.82% within one week (Figure 4C). For local social distancing measures, prohibiting public gatherings of more than 4 people achieved the most significant reduction effect, by reducing the overall onset risk by 45.91% (Figure 4C). Other effective measures have been the closure of bars, the closure of leisure venues, the restriction of restaurant capacity, and the strengthening of laboratory virus surveillance, enabling the overall onset risk to be reduced by 33.33%, 30.17%, and 16.26%, respectively (Figure 4).

**Figure 5:**
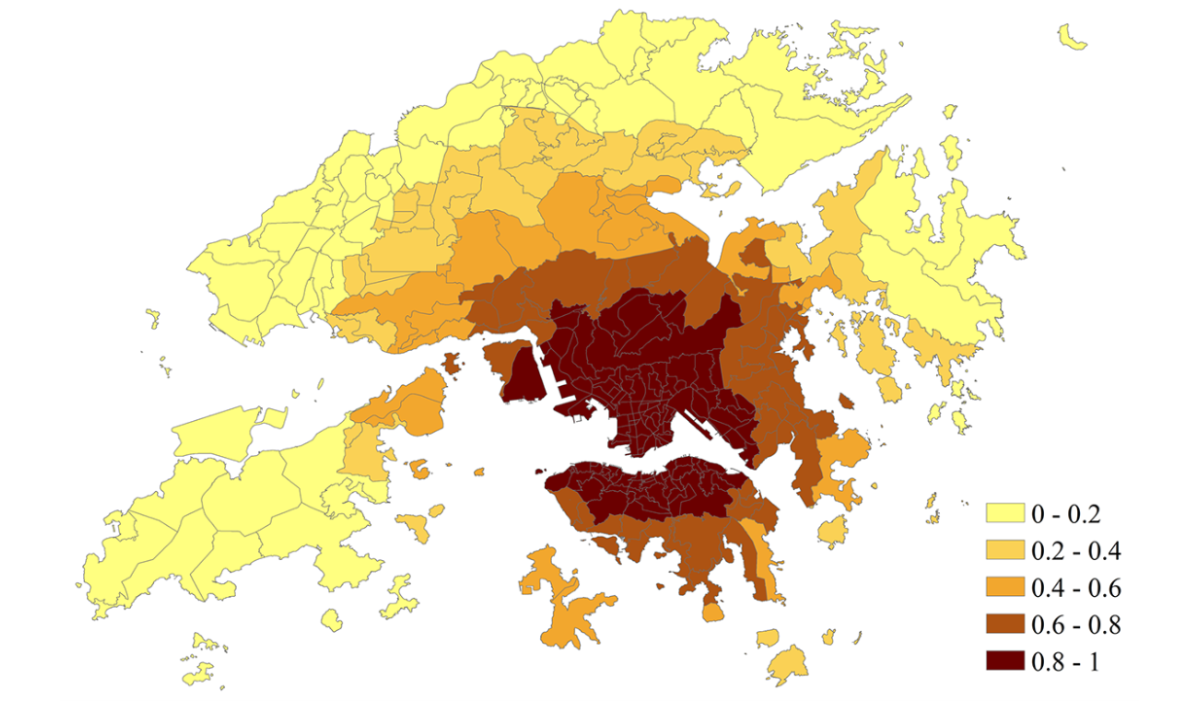
Predicted risk of COVID-19 symptom onset on January 23, 2021, for assisting the compulsory testing in Hong Kong.

**Figure 6:**
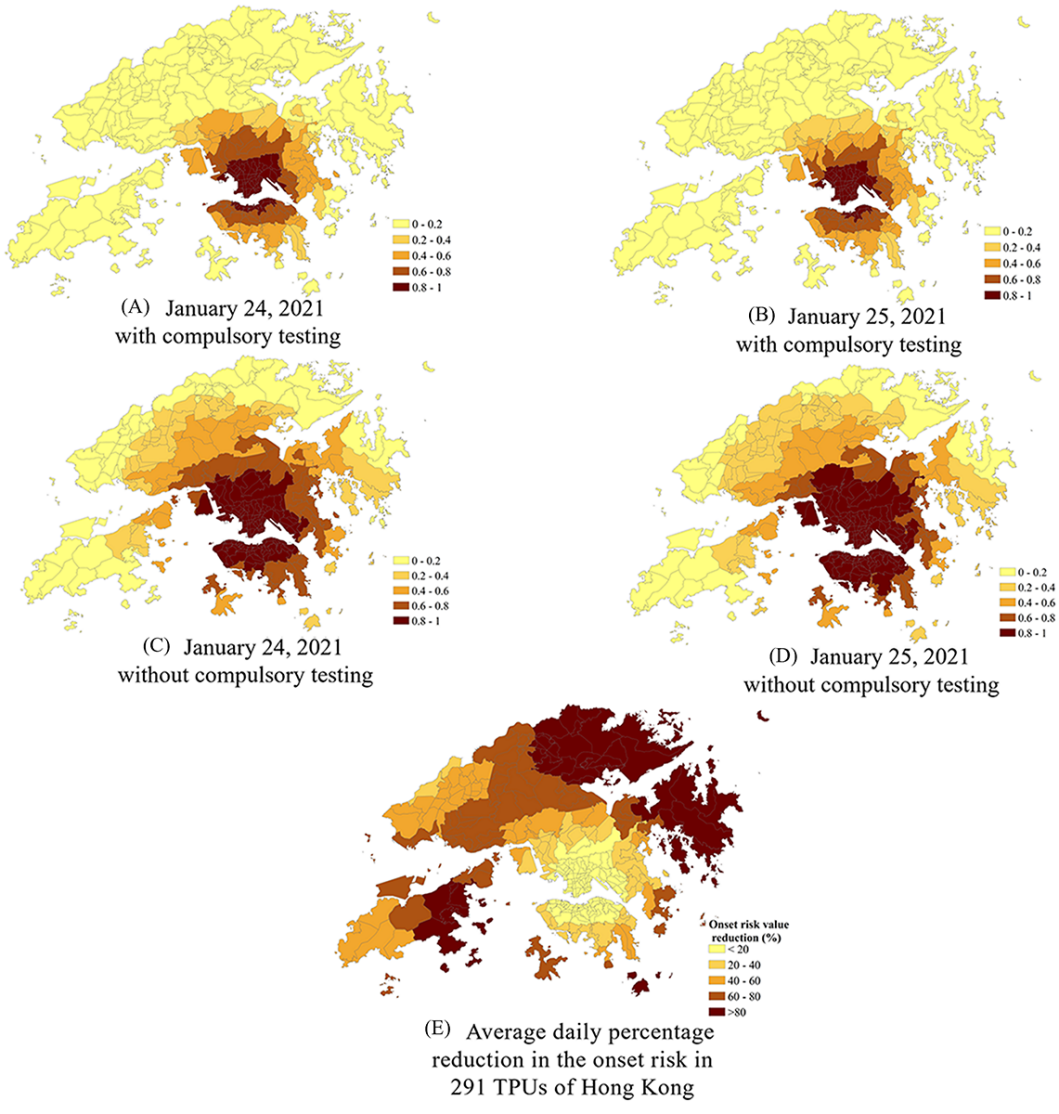
The risk of COVID-19 symptom onset under two scenarios (ie, with and without the compulsory testing measure) from January 24, 2021, to January 25, 2021. (A-D) The predicted symptom onset risk with (A-B) and without (C-D) the compulsory testing; (E) average daily percentage reduction in the onset risk in 291 tertiary planning units (TPUs) of Hong Kong in the compulsory testing scenario, compared with the noncompulsory testing scenario.

As the epidemic enters the normalization stage of prevention and control, COVID-19 response measures need to be more targeted than during the early stage of the pandemic. For the new response measures to be implemented, it is necessary to analyze the effects of new measures and assist in a more targeted implementation. For example, during the peak period of the recent fourth epidemic wave, the new measure—compulsory testing—was planned to be adopted from January 23, 2021. Before the implementation of the compulsory testing measures, based on the results of symptom onset risk prediction before January 23, 2021, “specified areas” with medium-high risk and “restricted areas” with high risk were quickly delineated by identifying the presence of TPUs within areas with medium-high onset risk and high onset risk (Figure 5). Moreover, in response to the public’s possible doubts about the controlling effects of the new measures, the simulation of the spatiotemporal effects of the new measures could also be provided, such as the simulation of the effect of the compulsory testing measure. The comparison of simulation results (Figure 6) showed that the compulsory testing not only suppressed the spread of SARS-CoV-2 in 291 TPUs but also reduced the onset risk related to each TPU. The onset risk was reduced by more than 80% in 42 (14.43%) TPUs and by more than 60% in 96 (32.99%) TPUs (Figure 6E; Table 1). Moreover, the compulsory testing was the most significant regarding the risk of those TPUs that had been originally at low or medium risk.

**Table 1:** The number and percentage of tertiary planning units (TPUs) at different reduction levels in the onset risk in the compulsory testing scenario, compared with the noncompulsory testing scenario.

Onset risk reduction (%)	Number of TPUs, n (%)
0-20	108 (37.11)
20-40	40 (13.75)
40 60	47 (16.15)
60-80	54 (18.56)
>80	42 (14.43)

### The Developed Web-Based Platform to Inform Governmental Agencies and Populations Based on the Symptom Onset Risk Predictions

The precise prevention and control in Hong Kong rely on effective government response and public cooperation. Based on the above onset risk prediction and analysis results, a web-based platform has been developed in this study. Through the services of this platform, the government and the public can obtain the prediction and analysis results of the risk of COVID-19 symptom onset within the next three days. Advanced deployment of epidemic prevention measures can be achieved especially when large-scale events are held. In addition, for the public cooperation, an intuitive understanding of the onset

risk predictions in the surrounding communities and potential travel communities helped in the adjustment of their daily activity plans in advance.

## Discussion

### Principal Findings

Global cities are taking swift actions in regard to building a sustainable postpandemic recovery [32]. COVID-19 is likely to stay with us as an endemic disease and cannot be eradicated globally or even eliminated regionally [33]. Thus, it is crucial for the recovery to identify resource mobilization needs, strengthening targeted measures and public cooperation in the new normalcy of long-term coexistence with SARS-CoV-2. Preemptively estimating the virus spread trend should become part of future epidemic prevention. Hence, in line with this aim, a spatiotemporal solution that supports more sustainable pandemic responses for returning to normalcy in the context of living with COVID-19 has been developed and is presented in this study. Since the COVID-19 epidemic outbreak, the proposed spatiotemporal solution has been supporting precise prevention and control in Hong Kong.

Firstly, the daily onset risk prediction results enables the spread of SARS-CoV-2 in communities to be timely assessed. It can therefore support the implementation of targeted and differentiated measures, especially at large events such as university entrance exams.

Secondly, under three main epidemiologic setting scenarios, high–onset-risk communities could be identified from the original daily onset risk prediction result and daily onset risk prediction enhanced by population distributions. It could limit the high–onset-risk communities in a small area in all three epidemiologic setting scenarios. The strictest COVID-19 response measures were only implemented in limited high–onset-risk communities to reduce the impact on normal social and economic activities.

Third, the effect of COVID-19 response measures already implemented by the government could be analyzed. These proven effective measures in this study have been playing a key role in Hong Kong’s epidemic prevention. This was important in the early stage when the characteristics of the spread of SARS-CoV-2 variants were not clear. This could help the government adjust previous measures in subsequent stages.

Fourth, the implementation of the new COVID-19 response measures could be assisted, such as the new compulsory testing measure in the fourth epidemic wave. Before this measure was implemented first, the high–onset-risk restriction area could be determined through onset risk prediction results.

Fifth, in response to the public’s possible doubts about the effects of the new COVID-19 response measures, the effects of the new COVID-19 response measures could be simulated. This is important with respect to the enhancement of policy transparency and also in building public trust in epidemic prevention.

Sixth, the prediction and set of analysis results related to onset risk have been integrated into a web-based visualized platform. Visualized information of spatiotemporal prediction and analysis enables the government and people to more easily and even more intuitively understand the nature of the potential spread of onset risk, leading to further understanding of symptom onset risk information related to each community. Logically, such understanding can better strengthen the appropriate response.

### Comparison With Prior Work

To the best of our knowledge, this is the first attempt to use spatiotemporal onset cases data to explore the spatiotemporal variations of onset risk at the urban-community scale in the context of the emergence of different variants since the epidemic outbreak. It can further support effective epidemic control at the urban-community level for returning to normal. The Space-Time Scan Statistical Analysis Method [34], the improved Susceptible Exposure Infection and Recovery model [35-37], the spatiotemporal Bayesian inference model [38], and the hierarchical clustering analysis [39] were used in the previous studies to investigate the impact of the spatiotemporal spread of COVID-19. Consistent with the conclusions of these studies, results of this study also show that precise epidemic prevention and control measures can help effectively control the epidemic. Similarly, when epidemic prevention and control measures are relaxed, high-risk areas will also expand significantly, especially in the early stages of the epidemic. However, the vast majority of previous modeling studies have focused on the national, state, and regional levels [40]. The strength of this study is that building an onset risk prediction model at the urban-community level can facilitate accurate and differentiated public health responses at the finer spatial scale [40]. This allows the vast majority of areas in the city to maintain normal social and economic activities while maintaining strict prevention and control measures in a very small number of areas. Meanwhile, it can also facilitate the more effective allocation of limited medical resources to the limited areas with high onset risk that need it. In addition, this study simulates and predicts the spatiotemporal spread of COVID-19 under different measures or population mobility based on the symptom onset risk model. Hence, epidemic prevention measures and people’s daily travel can be adjusted in advance in a timely manner. The proposed solution in this study can be used to support the enhancement need for community-based interventions and services in a multinational Delphi consensus to end the COVID-19 public health threat [41].

### Limitations

We acknowledge the following potential limitations: First, limited by the information of the current official data, the location information of the cases used in this study only includes community-level locations of residence. If more information about the work locations and visited locations are combined, urban-community–level onset risk associated with their spatiotemporal activities can be estimated more comprehensively. Second, the applied data of human mobility mainly include the traffic flow data on the main roads, which fails to more comprehensively reflect the population flow under multiple modes of transportation. In addition, various data that can reflect viral load of SARS-CoV-2 at different locations, such as sewage monitoring, can be incorporated into our proposed solution in the future.

### Conclusions

In the new normalcy of long-term coexistence with SARS-CoV-2, it is crucial to explore how to take the necessary response actions to reduce the impacts of the future epidemics and increase urban pandemic resilience for global cities [32]. Therefore, based on the enhanced prediction of COVID-19 symptom onset risk, a spatiotemporal solution has been proposed in this study. Such a solution has been used to support healthier and more sustainable pandemic responses in 291 communities of Hong Kong. The related web-based platform has served both the government agencies and Hong Kong residents. This solution not only assists global cities in responding to the future epidemic effectively but also helps to restore the social and economic activities and people’s normal lives. Based on this solution, we hope to make global cities more sustainable and hence promote the United Nations Sustainable Development Goals [42].

## Appendix

Multimedia Appendix 1 The technical details about the enhanced weighted kernel density estimation (WKDE) model and the Geodetector.

## Abbreviations

TPU: tertiary planning unit
WKDE: weighted kernel density estimation
