# Supplementary material for: A Spatiotemporal Solution to Control COVID-19 Transmission at the Community Scale for Returning to Normalcy: COVID-19 Symptom Onset Risk Spatiotemporal Analysis
Source: JMIR Public Health Surveill. 2023 Jan 6;9:e36538. doi: 10.2196/36538 (PMC9829029; doi:10.2196/36538)
Supplement: Multimedia Appendix 1 [file publichealth_v9i1e36538_app1.docx]

Supplement for “A Spatiotemporal Solution to Control COVID-19 Transmission at the Community Scale for Returning to Normalcy: COVID-19 Symptom Onset Risk Spatiotemporal Analysis”

# An enhanced urban-community-scale WKDE model for predicting the onset risk of COVID-19 symptoms: the technical details.

From the main paper, the main improvement of the enhanced urban-community-scale WKDE model was seen as the historical existence likelihood of the infection in a spatial location at the step ii) and is formulated [10, 14-16]:

 (1)

where P*_Infection_*(*S*, *t_i_*) is the probability of any infected person infecting others in a random location *S* in the city on day *t_i_* [10, 14-16]. *L_j_* is the *j*-th location among the places where onset cases remained [10, 14-16]. *P_Infection_*(*L*, *t_i_*) denotes the probability that one onset case was infected on day *t_i_* in location *L* [10, 14-16]*.* *K_h_*(*S* – *L_j_*) denotes a Gaussian kernel between locations *S* and *L_j_* [10, 14-16]*.* The values of *P_Infection_* (*L_j_*, *t_i_*), *K_h_* (*S-L_j_*) and *h* have been determined in earlier model procedures [10, 14-16]. R_t_(t_i_) denotes the reproductive number for local cases in the city on day *t_i._* V_E_(*t_i_*) denotes the vaccine efficiency on day *t_i_*; V_P_(*t_i_*) is the proportion of the population who, on day t_i_, have been fully vaccinated [10, 14-16]*.*

A_t_(t_i_) denotes the factor about the imported case risk caused by imported passengers in the city on day t_i_, calculated as follows [10, 14-16]:

 (2)

where a_k_ denotes the daily passenger arrivals in the city on day ti on day *t_k_* prior to *t_i_*.

*M_intra_TPU_*(*S*, *t_i_*) denotes a human mobility factor within a TPU containing location *S* on day *t_i_*, calculated as follows [10, 14-16]:

 (3)

where *X_k_* denotes the daily traffic flow within the TPU containing location *S* on day *t_k_* prior to *t_i_*.

*M_interTPU_*(*S*, *t_i_*) denotes a human mobility factor from other TPUs to the TPU containing location *S*, calculated as follows [10, 14-16]:

 (4)

where *Y_k_* denotes the daily traffic flow from other TPUs to the TPU containing location *S* on day *t_k_* prior to *t_i_*.

In particular, in the simulation of onset risk prediction with the compulsory testing measure implemented between 23 January 2021 to 25 January 2021, the daily human mobility in that high-onset-risk restricted area was defined as 0. In the simulation of onset risk prediction without the compulsory testing measure, the daily human mobility in the restricted area was assumed to be the previous normal human mobility.

Furthermore, a popular indicator for the prediction accuracy of the KDE model is the hit rate [10, 14-16]. The hit rate is defined as the percentage of all incidents (e.g., crimes) at a later Time 2, that are captured by the identified hotspots created from data at an earlier Time 1 [10, 14-16]. Following the idea of a hit rate, the accuracy of the enhanced urban-community-scale WKDE model is set as the percentage of all actual onset cases on the date of prediction that occur in the areas with predicted onset risk higher than 0.8 (‘hotspots’) [10, 14-16].

The population distributions in different communities were further used to enhance the original onset risk and is formulated as follows [10, 14-16]:

 (5)

where P*_P-onset_*(*S*, *t_z_*) is the onset risk value enhanced by population distribution in a random location *S* in the city on day *t_z_* [10, 14-16]. *P_onset_*(*S, t_z_*) denotes the likelihood that at least one person infected by a onset case at location *S* develops clinical symptoms on day t_z_ [10, 14-16]. The value of *P_onset_*(*S, t_z_*) has been determined by the WKDE model in step iii). T_i_ is the population of the i-th community where the random location *S* located in. $\sum_{k=1}^{T} Population(k)$ denotes the total population in all communities. Similar to the original onset risk, the P*_P-onset_* (*S*, *t_z_*) was also standardized to a value between 0 and 1.

# Geodetector

To explore the spatial association between the potential factors (i.e., mobility) and the predicted risk of COVID-19 symptom onset, the Geodetector was used to determine the degree of the determinant power of the potential factors in the spatial stratified heterogeneity of the COVID-19 symptom onset risk. Thus, the factor detection in the Geodetector is conducted by q-statistic [10, 14-16]:


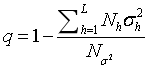
 (6)

where q is the explanatory power of factor *X* on the spatial heterogeneity of the factor *Y* (i.e., the predicted risk of COVID-19 symptom onset) [10, 14-16]. The value of the q-statistic is between [0, 1], and its value ranges from 0 to 1 [10, 14-16]. The larger the value of q, the stronger the effect of the factor *X* on the factor *Y* [10, 14-16]. The whole study area was divided into L layers, which are denoted by h = 1, 2, ..., L. N and N_h_ are the number of communities in the whole study area and strata h, respectively [10, 14-16].
